# The Relationship Between Neurocognitive Functioning and Occupational Functioning in Bipolar Disorder: A Literature Review

**DOI:** 10.5964/ejop.v12i4.909

**Published:** 2016-11-18

**Authors:** Walace Duarte, Rodrigo Becerra, Kate Cruise

**Affiliations:** a School of Psychology and Social Sciences, Edith Cowan University, Joondalup, Western Australia, Australia; EJOP Senior Editor, The Netherlands

**Keywords:** bipolar disorder, neurocognition, functioning, occupation, employment

## Abstract

Neurocognitive impairment in Bipolar Disorder (BD) has been widely reported, even during remission. Neurocognitive impairment has been identified as a contributing factor towards unfavourable psychosocial functioning within this population. The objective of this review was to investigate the association between neurocognitive impairment and occupational functioning in BD. A literature review of English-language journal articles from January 1990 to November 2013 was undertaken utilising the PsychINFO, Scopus and Web of Knowledge databases. Studies that made specific reference to occupational outcomes were included, and those that reported on global psychosocial measures were excluded. Majority of the papers reviewed (20 out of 23) identified an association between neurocognitive impairment (particularly in executive functioning, verbal learning and memory, processing speed and attention) and occupational functioning. Several methodological issues were identified. There was a discrepancy in the measures used to assess neurocognitive function across studies and also the definition and measurement of occupational functioning. The clinical features of the samples varied across studies, and confounding variables were intermittently controlled. The review focused on English-language papers only and hence there is a bias toward the Western labour market. These limitations therefore influence the generalizability of the interpreted findings and the reliability of comparisons across studies. Neurocognitive impairment in BD appears to play a role in occupational outcomes. The findings of this review highlight the challenges for future research in this area, particularly in the measurement of neurocognitive and occupational functioning. Incorporating neurocognitive interventions in the treatment of BD, which has traditionally focussed solely on symptomatic recovery, may advance the vocational rehabilitation of these patients.

Bipolar Disorder (BD) is a chronic mood disorder characterised by episodes of elevated mood, typically alternating with episodes of depression. The DSM-V (American Psychiatric Association, 2013) identifies four subtypes: Bipolar Disorder I (BD I), Bipolar Disorder II (BD II), Cyclothymia and Bipolar Disorder not otherwise specified. BD I is characterised by manic or mixed (including psychotic) episodes, normally with major depression. BD II is characterised by moderate elevations in mood (hypomania) and episodes of major depression. BD affects between 2.5-3.3% of Australian Adults (Zutshi, Eckert, Hawthorne, Taylor, & Goldney, 2011) and has been identified as one of the most expensive of all mental illnesses. In 2004, the excess cost of BD in Australia was estimated between $3.97 and $4.95 billion (Fisher, Goldney, Dal Grande, Taylor, & Hawthorne, 2007). BD is associated with a substantial risk of suicide; with the lifetime for BD at least 15 times that of the general population (American Psychiatric Association, 2013). More than half those diagnosed with BD have comorbid alcohol use disorders, which further increases the risk of suicide and BD is also associated with high rates of comorbid anxiety, ADHD, and impulse control disorders (American Psychiatric Association, 2013).

The multi-dimensional nature of BD (heterogeneous, episodic, recurrent) pose challenges for effective diagnosis, treatment and management (Anderson, Haddad, & Scott, 2012). The treatment of BD has traditionally targeted alleviating the effects of depression and mania. Even when symptomatic recovery is attained, significant functional impairments often persist (Tohen et al., 2000). It has been suggested that measures of functional outcome provide a more reliable indicator of response to treatment in BD than clinical measures such as a reduction in symptoms (Keck, 2004).

Michalak and Murray (2010) define *psychosocial functioning* as a person’s ability to perform activities of daily living and to engage in meaningful interpersonal relationships. Impairment in psychosocial functioning is a defining feature of BD (American Psychiatric Association, 2013) and has been widely reported in the literature, even during periods of remission (MacQueen, Young, & Joffe, 2001; Sanchez-Moreno et al., 2009). Difficulty maintaining established relationships within the family and forming new relationships outside of the family have been reported among individuals living with BD (Elgie & Morselli, 2007). In addition, individuals living with BD report reduced quality of life (Michalak, Yatham, & Lam, 2005) and health related quality of life (Dean, Gerner, & Gerner, 2004). Occupational functioning is an important aspect of psychosocial functioning that is also reduced among individuals living with BD.

Occupational difficulties that have emerged from the literature include prevalent long term unemployment, difficulty sustaining employment, reduced work efficiency and absenteeism (Dean et al., 2004). These difficulties appear to increase over time and in response to multiple periods of acute illness (Zimmerman et al., 2010). Research indicates that individuals living with BD experience significantly higher levels of unemployment compared with both the general population and those with unipolar depression (Shippee et al., 2011; Waghorn & Lloyd, 2005). In their review of the literature, Marwaha, Durrani, and Singh (2013) observed that over time, people with BD who remain employed move into less demanding work roles. Issues around disclosure and stigma in the workplace (Michalak, Yatham, Maxwell, Hale, & Lam, 2007) add another level of complexity to these concerns.

Factors that have been associated with impaired psychosocial functioning in BD include current symptomatology, particularly depressive symptoms (Bauer et al., 2001; Martino et al., 2009; Simonsen et al., 2010), previous hospitalisations and mixed episodes (Rosa et al., 2009), number of medications taken (Martínez-Arán et al., 2007) and genetics (Levy & Manove, 2012). Neurocognitive impairment is another factor that has emerged from the research and is thought to undermine psychosocial functioning in BD (Baune, Li, & Beblo, 2013; Tabarés-Seisdedos et al., 2008).

Although diagnostically known as a mood disorder, BD patients also experience neurocognitive impairment (Robinson et al., 2006). Reviews of the literature suggest that the cognitive deficit in BD is present among both younger and older patients (Cahill, Green, Jairam, & Malhi, 2007; Delaloye et al., 2009) and occurs across subtypes [i.e., in both BD I and BD II: (Bora, Yücel, Pantelis, & Berk, 2011)] with increased impairment evident in BD I (Sole et al., 2012; Torres, Solé, Vieta, & Martínez-Arán, 2012). Neurocognitive impairment has been reported during both acute mood and euthymic states (Andreou & Bozikas, 2013; Depp et al., 2012a; Kurtz & Gerraty, 2009; Wingo, Harvey, & Baldessarini, 2009), with significant deficits identified for attention, processing speed, memory and executive functioning (Torres, Boudreau, & Yatham, 2007).

It is reasonable to suggest that neurocognitive functioning subserves not only complex, abstract processing but also daily basis processing; therefore deficits in this area would have ramifications for occupational functioning. For example, memory is utilised in skill acquisition, and attention is required for job performance. This suggests that a substantial component of successful occupational outcomes rest upon satisfactory neurocognitive performance.

Reviews to date have looked at the relationship between neurocognitive impairment and psychosocial functioning in general, incorporating research articles that have primarily utilised global measures of functioning (Depp et al., 2012a; Wingo et al., 2009). Two recent reviews focussed on the predictors of employment in BD. Gilbert and Marwaha (2013) identified cognitive deficits, depression and level of education as predictors of employment in BD. A meta-analysis conducted by (Tse, Chan, Ng, & Yatham, 2014) identified a relationship between cognitive performance and favourable employment outcomes in BD, and mediating effects of years of education, course of illness, and symptomatology. Executive functioning and verbal memory were highlighted as particularly relevant cognitive domains underlying occupational functioning (Gilbert & Marwaha, 2013; Tse et al., 2014).

The following literature review will focus exclusively on the association between neurocognitive impairment in BD and occupational functioning, incorporating a larger body of literature on this topic. The term occupational functioning has been used so as to not only include studies that focus on employment, but also other related functions that contribute to employment, such as work skills and pre-employment activities. It is expected that by examining occupational outcomes this will provide a broader perspective in this area of functioning in BD.

Employment has been identified as a vital contributor for the wellbeing and quality of life for those with BD (Eklund, Hansson, & Ahlqvist, 2004; Nordt, Müller, Rössler, & Lauber, 2007). This highlights the importance of occupational activity for individuals living with BD, and highlights the potential for occupational functioning as a target for intervention. It is anticipated that the findings from this review will contribute to the knowledge in this field, and inform practitioners working directly in the rehabilitation of BD patients.

## Methods

A search of the PsychINFO, Scopus and ISI Web of Sciences databases was conducted for relevant English-language, peer-reviewed original journal articles, dating from January 1990 to November 2013. Research into neurocognitive functioning within the BD population appears to have received more rigorous attention over the last 10-15 years (Kurtz & Gerraty, 2009) and therefore the aforementioned time frame was selected.

A combination of the following three sets of terms were used in the search, joined by "AND" operators. The first set identified the BD population; "Bipolar Disorder", "Bipolar I", "Bipolar II", "Affective Disorder", "Mood Disorder", "Mania" or "Manic Depression". The second set identified the neurocognitive component; "Neurocogniti*", "Neuropsych*", "Cogniti*", "Executive" or "Intellect*". The third group identified occupational functioning; "Vocation", "Employ*", "Work", "Job", "Occupation*" or "Function*".

Results of this search were first screened for relevance via the title, then by inspection of the abstract. References lists were also searched for relevant articles. Any uncertainty regarding the relevance of an article was taken to the second author for a decision on inclusion or otherwise. A total of 46 articles were identified by this process.

These 46 articles were then examined for specific reference to the relationship between neurocognitive factors and occupational functioning. Twenty-one articles were excluded, mainly as a result of the use of global measures of functioning (for example; Global Assessment of Function and the Social Adjustment Scale) and also a result of failure to specifically identify occupational functioning.

The effect sizes for significant results are reported in Table A1. For studies where effect size data were not available, effect sizes were calculated and converted to Cohen’s *d* or *f^2^* (for regression). The standardised mean difference (*d*) was calculated for studies involving between groups designs. For studies involving Chi-square and correlational outcome data, χ^2^ and *r* values were converted to *d.* Cohen’s *d* was calculated for studies utilising regression analysis that provided β values in conjunction with the standard deviation of the dependent variable and sample size. For regression where *R*^2^ values were reported, *R*^2^ was converted to *f*^2^.

## Results

Twenty-three articles were selected for inclusion in the current review (see Table A1). With no papers published before 2004, and 17 since 2010, it is clear that research into the association between neurocognitive functioning and occupational functioning in BD is a relatively new field that is gaining increasingly more interest.

The majority of studies (16) utilised outpatient participants and were primarily conducted in the USA (12) and Spain (9), with single representations from England, Canada, Ireland and Norway. The following section will examine the assessment of neurocognitive functioning, the measurement of occupational functioning, and then the relationship between these two factors.

### Neurocognitive Assessment

The majority of studies (20) administered a battery of objective neurocognitive measures, primarily covering the domains of executive functioning, verbal memory, attention, and processing speed. Exceptions included Gilbert et al. (2010) who used a self-report assessment and a clinical interview to collect information regarding memory and concentration and Altshuler et al. (2007) who used a structured interview to assess executive functioning. Schoeyen and colleagues (2013) utilised a neurocognitive test battery to measure participants Full Scale IQ and premorbid intellectual functioning, arguing that the various neurocognitive domains reflect general intellectual ability. O'Shea et al. (2010) administered ecologically valid cognitive tests (daily, real-life tasks) to measure their participant’s attention, memory and executive functioning.

### Measures of Occupational Functioning

The methods used to assess occupational functioning varied widely between studies. Most commonly, participants were categorised based on employment status (full-time, part-time or otherwise). Another method was to measure occupational functioning or adaptation, using a particular instrument or by categorising participants into a "good" or "poor" group based on occupational adaption. Finally, a small group used miscellaneous methods, beyond these broad descriptions.

Nine studies (Altshuler et al., 2007; Depp et al., 2012b; Dickerson et al., 2004; Dickerson et al., 2010; Gilbert et al., 2010; Mora, Portella, Forcada, Vieta, & Mur, 2013; Mur, Portella, Martínez-Arán, Pifarre, & Vieta, 2008; Mur, Portella, Martínez-Arán, Pifarre, & Vieta, 2009; Ryan et al., 2013) measured occupational functioning by categorising participants into groups based on their employment status. Of these studies, the most common method involved creating a ‘working vs not working’ dichotomy (Altshuler et al., 2007; Depp et al., 2012b; Dickerson et al., 2010; Gilbert et al., 2010; Mur et al., 2009; Ryan et al., 2013). Other studies created a third grouping with the inclusion of a "part-time" or "retired/disabled" group (Dickerson et al., 2004; Levy, Medina, & Weiss, 2013; Mora et al., 2013; Mur et al., 2008). Definitions also varied within this employment status category, with Ryan et al. (2013) and Dickerson et al. (2010) describing their "working" group as having attained full-time employment, whilst others (Altshuler et al., 2007; Depp et al., 2012b; Gilbert et al., 2010; Mora et al., 2013; Mur et al., 2008; Mur et al., 2009) including participants who were part-time employed.

A variety of formal assessment measures were also employed among studies to assess occupational functioning, including: the Longitudinal Interval Follow-up Evaluation-Range of Impaired Functioning Tool (Godard, Grondin, Baruch, & Lafleur, 2011), the Life Functioning Questionnaire (Bearden et al., 2011), the Vocational Status Index (Wingo, Baldessarini, Holtzheimer, & Harvey, 2010), Functioning Assessment Short Test (Bonnín et al., 2010), and the Social and Occupational Functioning Assessment Scale (O'Shea et al., 2010).

Martínez-Arán et al. (2004) examined occupational functioning, but by dividing participants into two groups - good or poor - based on whether they worked at a good/acceptable level of functioning or otherwise. However, in subsequent papers (Martínez-Arán et al., 2007; Tabarés-Seisdedos et al., 2008; Torrent et al., 2006), these researchers measured occupational outcomes by way of occupational adaptation. They divided participants into "good" or "poor" occupational adaptation groups, determined by a good/acceptable level of functioning most of the time, versus those not working or exhibiting difficulties in their jobs.

Six papers employed other means of assessing occupational outcomes. Burdick, Goldberg, and Harrow (2010) measured work disability with the Strauss-Carpenter work functioning rating scale; Bowie et al. (2010) examined work skills using the Specific Level of Functioning Scale; Schoeyen et al. (2013) used receipt of a disability pension to represent poor occupational outcomes; Bonnín et al. (2014) divided work participants into "good" or "poor" work adjustment based on their participation in full- or part-time employment, or otherwise; and Murtagh et al. (2010) considered participants work history for the last three years to create two groups - those that had worked for at least six months during that time and those that had not.

### The Relationship Between Neurocognitive Functioning and Occupational Functioning

Twenty studies identified a relationship between neurocognitive functioning and occupational functioning in BD. The major finding reported among these studies was that neurocognitive impairment was associated with diminished occupational functioning.

Just over half (12) of these studies utilised euthymic participants, and the same number of studies also excluded individuals with recent substance abuse or dependence. Eight studies controlled for the effects of medication. Nineteen of these studies incorporated solely BD populations (with or without healthy controls), whilst four included other diagnostic categories such as schizophrenia (Bowie et al., 2010; Godard et al., 2011; Murtagh et al., 2010; Tabarés-Seisdedos et al., 2008). Only Torrent et al. (2006) differentiated between BDI and BDII; noting that the relationship between neurocognitive and occupational functioning was evident for both subtypes.

Of the 23 articles, 14 were cross-sectional, six were longitudinal and two were post-hoc design. Longitudinal studies ranged from three months (Levy, Medina, & Weiss, 2013) to 15 years (Burdick et al., 2010). The most common neurocognitive domains implicated in this relationship were: executive functioning (12), verbal learning/memory (8), processing speed (5) and attention (5). Depp et al. (2012b) and Bowie et al. (2010) did not identify specific domains, instead reporting on overall neurocognitive functioning.

Bowie et al. (2010) noted that the relationship between neurocognitive functioning and work skills in BD was indirect and was mediated by adaptive and social competence. The authors defined adaptive competence as the instrumental skills important for functioning independently, and social competence as the linguistic and verbal behaviours essential for communication.

Of the three papers that did not find an association between neurocognitive functioning and occupational functioning, Dickerson et al. (2010) noted that the processing speed domain approached significance for work adjustment in their study, whilst Wingo et al. (2010) reported that their participants differed on various neurocognitive measures, though not to a significant level. Schoeyen et al. (2013) failed to find a relationship between overall neurocognitive functioning, as measured by IQ, and receipt of a disability benefit.

## Discussion

The aim of this paper was to investigate the association between neurocognitive and occupational functioning in BD. Of the papers reviewed, most (20) identified a relationship between impaired neurocognitive functioning and reduced occupational functioning in BD. This is consistent with the findings of a recent systematic review and a meta-analysis which considered predictors of employment in BD (Gilbert & Marwaha, 2013; Tse et al., 2014). The current review identified a number of neurocognitive domains that appear to be particularly sensitive to changes in occupational functioning including: executive functioning, verbal memory, processing speed and attention. Although over half (14) of these papers were cross-sectional, seven longitudinal studies identified that the relationship between neurocognitive impairment and reduced occupational functioning was stable over time, and that neurocognitive assessment may provide prognostic information regarding occupational functioning in BD.

Although the relationship between neurocognitive impairment and reduced occupational functioning appears to be stable over time, the current review indentified that certain cognitive domains are more sensitive to changes in occupational functioning in longitudinal studies compared to cross-sectional studies. For example, reduced performance on measures of verbal and working memory were found to be more strongly associated with reduced occupational functioning overtime, and the strength of these effects were considered to be medium to large for both verbal and working memory. Reduced performance on measures of verbal memory were also implicated in reduced occupational functioning in cross-sectional studies suggesting that verbal memory is an important cognitive domain over both short and long periods. Although working memory appeared to be an aspect of executive function implicated in longitudinal studies, measures of executive function associated with cross-sectional studies were somewhat variable. For example, verbal fluency and inhibition were more sensitive to changes in occupational functioning among follow-up studies and the strength of the effect varied substantially. One study reported a small to medium effect size for verbal fluency (Ryan et al., 2013), whereas another study reported a large effect size (Godard et al., 2011). Differences in methodology may underlie such discrepancies, and further information regarding the strength of the relationship between measures of executive function and occupational functioning is required, given that effect sizes could not be calculated for a number of studies.

### Clinical Variables

A number of studied identified residual depression as an important predictive factor of employment among BD populations (Gilbert & Marwaha, 2013; Tse et al., 2014). Out of the studies reviewed, approximately half (12) utilised euthymic participants in an attempt to control for the effects of depression. It should be noted though that studies varied in how euthymic populations were defined. Generally (in 8 instances), a Hamilton Depression Rating Scale score below 8 and a Young Mania Rating Scale (YMRS) score below 6 were used to characterise euthymic states, usually stipulated over the last 3-6 months (7). There were variations from this, for example, in two cases a YMRS score below 8 was used, and in another two cases euthymia was prospectively verified over a period of 6 months. Although establishing a standard definition of euthymia remains a challenge (a meta-analysis of cognitive deficits in euthymic BD patients identified 23 different descriptions [Robinson et al., 2006]), Torres et al. (2007) suggest that minimal mood rating scores should be employed. In light of the potential effects of mood on occupational functioning in BD, future research in this area should ideally include euthymic populations, defined by stringent criteria.

A brief discussion is warranted regarding factors that can influence neurocognitive functioning in BD. There is debate as to whether psychotropic medication, particularly anti-psychotics, polymedication regimes and high dosages have an impact on cognitive function with some authors stating that the side effects of medication influence cognitive functioning (Balanzá-Martínez et al., 2010; Torres, Solé, Vieta, & Martínez-Arán, 2012); whilst other authors argue that the effects of medication on cognitive functioning in BD are minimal (Kurtz & Gerraty, 2009; Robinson et al., 2006; Torres et al., 2007). Given the heterogonous nature of BD, medication regimes will vary widely, therefore attempts to control for the effects of medication will ensure that the potential for neurocognitive side-effects are minimised.

Another factor which has the potential to impact on cognitive function, and hence the relationship between neurocognitive and occupational functioning, is comorbid substance abuse. Balanzá-Martínez et al. (2010) noted in their review that neurocognitive functioning in BD is not only impaired by current and recent alcohol use, but even following a period of abstinence from alcohol. In the current review, those studies that excluded participants based on drug and alcohol dependence or abuse (13) varied in their defined periods of abstinence, with periods ranging from 12 months to no current substance abuse/dependence issues (3). The longer participants can abstain from alcohol and illicit drugs, the less likely these substances will have an effect on neurocognitive functioning. However, it is noted that there is a high level of comorbidity between BD and substance abuse and/or dependence, alcohol in particular (American Psychiatric Association, 2013) and excluding or controlling for current substance use may influence the generalizability of research findings in the area. Future research in the area may benefit from reporting the results with and without statistically correcting for substance use in order to examine the impact of comorbid substance abuse more closely.

Wingo et al. (2009) identified other factors known to influence neurocognitive functioning in BD including age, education, premorbid IQ, and course of illness or chronicity factors. History of psychosis was identified as factor contributing to poor neurocognitive function (Wingo et al., 2009) and also poor occupational functioning (Levy et al., 2013). There appears to be a complex relationship between these mediating factors and the outcome variables for neurocognitive and occupational functioning. For example, Wingo et al. (2010) reported that the relationship between neurocognitive and functional outcomes was reduced after adjusting for education and residual mood symptoms. Similarly, Schoeyen et al. (2013) failed to find a correlation between either premorbid or current IQ with receipt of either a full-time or part-time disability benefit (an indicator of poor occupational functioning), though a significant association emerged when clinical variables such as the number of hospitalisations for depressive episodes and illness duration were considered.

### Methodological Issues and Limitations

#### Neurocognitive Assessment

The first methodological issue relates to the assessment of neurocognitive functioning. The domains of cognitive functioning assessed varied across studies, for example executive functioning (16 studies), verbal learning/memory (16), attention (15) and processing speed (11) were the most commonly measured domains. Six studies included a measure of visual memory, and two included a measure of motor control and co-ordination.

The majority (21) of studies reviewed utilised a battery of standardised objective neurocognitive tests; however the assessments comprising each ‘battery’ varied widely across studies. For example, the Trail Making Test B (TMT-B) (11 papers), Controlled Oral Word Association Task (FAS) (11) and the Wisconsin Card Sorting Test (WCST) (11) were the most commonly used assessments of executive functioning, however, only four studies (Bonnín et al., 2010; Bonnín et al., 2014; Ryan et al., 2013; Tabarés-Seisdedos et al., 2008) used all three measures in the same study. There was also inconsistency surrounding the cognitive domains that certain assessments were alleged to be measuring. For example, although the TMT-A is widely used as a measure of processing speed and the TMT-B task is thought to provide a measure of cognitive flexibility, and hence a measure of executive function, the TMT-B task was used as both a measure of attention and executive function among the studies reviewed. Future research would benefit from a standardised battery of neuropsychological assessment and efforts have been made to develop a standard neurocognitive assessment battery specifically for BD research (Yatham et al., 2010). Yatham and colleagues identified executive functioning, verbal learning/memory, attention/vigilance, visual learning, working memory, and speed of processing as important cognitive domains to be investigated in BD research and proposed the use of the TMT-B, WCST, and Stroop Colour Word Association Tests as the most valid measures of executive functioning. Yatham et al. (2010) note that a standard battery of neurocognitive assessments for BD, offer researchers not only the opportunity to meaningfully interpret and compare results across studies, but also the ability to pool and analyse results over a larger sample size (e.g. meta-analysis). The authors emphasise though that in the absence of psychometric validation and further research, these are only introductory steps towards compiling a standard neurocognitive assessment battery for BD.

The association between neurocognitive functioning and occupational functioning also emerged in a small group of research which utilised non-standardised neurocognitive assessments (Altshuler et al., 2007; Gilbert et al., 2010; O'Shea et al., 2010). O'Shea et al. (2010) and Gilbert et al. (2010) offer particularly unique perspectives on this matter. O'Shea et al. (2010) administered ecologically valid neurocognitive tests, indicating that there is no definitive relationship between successful daily functioning and the results of standardised cognitive tests. Gilbert et al. (2010) used a self-report assessment of neurocognition, specifically targeting concentration. Both Gilbert et al. (2010) and O'Shea et al. (2010) suggested that utilising ecologically valid measures of neurocognitive functioning that reflects performance in a specific context or area of functioning (e.g., occupational functioning), may help to improve predictions of employment trajectory. Burdick, Endick, and Goldberg (2005) report in their study that although self-reported cognitive impairments failed to correlate with objective neurocognitive assessments, the former measures do provide practitioners with valuable information, particularly regarding treatment adherence. Further research into the ecological (face) validity of neurocognitive assessments, and the utilisation of either subjective or objective measures, in light of occupational activity, is warranted.

#### Occupational Functioning

The second methodological issue concerns the definition and measurement of occupational functioning, which varied widely between papers reviewed. Classifying occupational status as ‘working’ or ‘not working’ provided the strongest indicator of occupational functioning across studies and consequently was the most common measure employed by studies in this review. Variations within this dichotomous approach were noted, with part-time work sometimes excluded from the ‘working’ group (Dickerson et al., 2010; Ryan et al., 2013). Occupational functioning was also measured using a range of instruments, with one group of researchers measuring occupational adaptation, whilst others considered work disability, work skills, and even participant progress towards engagement in vocational services as measures of occupational functioning. Schoeyen et al. (2013) offered a novel perspective by using receipt of a part- or full-time disability benefit as a measure of poor occupational functioning. This method poses some important limitations, for example, some participants may have been working and receiving a (particularly part-time) disability benefit. Tse and colleagues (2014) suggest that other indicators of occupational functioning should be considered, such as absenteeism, job satisfaction and whether a job matches the person’s qualifications or skills. All these indicators reflect the breadth of occupational outcomes, and highlight the extent of occupational impairment in BD. This diversity in the way in which occupational functioning is defined and measured limits the opportunity for comparison between studies.

Information regarding occupational functioning was collected by variety of methods including self-report, clinician-report and performance-based measures. All methods of measurement are limited in some form, for example, self-report bias, inter-rater variability in clinician-based measures, and the narrow representation of functioning in performance-based measures (Baune et al., 2013). In their review of psychosocial outcomes in BD, MacQueen et al. (2001) noted that functional impairments were less when participants were defined as employed or not, than when self-reported effects of illness on psychosocial functioning were identified. The authors go onto explain that performance-based measures can miss more subtle deficits in the participants functioning, and also ignores the participants view of their own level of functioning. Depp et al. (2012b) on the other hand, reported in their meta-analysis that real world outcomes, such as employment and performance-based measures of functioning demonstrated stronger associations with cognitive abilities than self-report and clinician-rated measures. Furthermore, Wingo et al. (2009) report that indicators such as employment may provide a more objective measure of real-world functional performance. In summary, although there are limitations to the various methods of measuring occupational functioning, an objective performance-based indicator such as employment status may provide a real-world measure of occupational functioning, which also facilitates comparisons made between studies.

A potential bias regarding occupational functioning is noted with regards to the inclusion criteria for this review. Limiting the search to English-language studies restricts cross-cultural comparisons, and also tends to reflect Western labour markets. This may pose different consequences for occupational functioning (e.g. employment opportunities) to other economies.

### Methodological Limitations

Sample size and therefore statistical power varied across studies, with only 12 papers involving samples greater than 100 participants. Differences in sample size and characteristics make it difficult to generalize interpretations across studies. The clinical samples comprising each study also carried widely. Some studies did not distinguish between BD I and BD I and others incorporated other diagnoses such as schizophrenia. It is therefore difficult to generalise across studies given the heterogeneous nature of BD. The majority of the studies reviewed were cross-sectional, and this also limits the long term generalisations and inferences regarding causal directions for the relationship between cognitive and occupational functioning in BD.

### Future Directions and Implications

Despite these limitations the research at hand does seem to suggest that neurocognitive impairment is associated with (and may predict) diminished occupational functioning in BD, and this has important implications for rehabilitation practitioners. Given that most jobs require a degree of memory and attention, it follows that interventions targeting neurocognitive functioning may help to improve occupational functioning. Harvey et al. (2010) suggested that cognitive remediation interventions (cognitive training and exercises aimed at improving neurocognitive functioning), which have been successfully implemented with schizophrenia patients for the last four decades, may also assist BD populations. Indeed, in the first study of its kind utilising a homogenous BD sample, Deckersbach et al. (2010) reported that cognitive remediation, in combination with CBT aimed at depressive symptoms, resulted in improved occupational functioning, which was also associated with improvements in executive functioning. More recently, Torrent et al. (2013) evaluated the efficacy of a functional remediation program among a sample of euthymic patients with BD. This intervention involved exercises to improve memory, attention, and executive function in order to enhance daily routine. These authors reported that the intervention was associated with significant improvements in functional outcomes compared to treatment as usual, however functional remediation was no more effective than psycho education. A randomised controlled trial conducted by Demant et al. (2013) also demonstrated significant improvements neurocognitive and functional outcome measures as a result of undertaking a group-based cognitive remediation program. In their review of studies where cognitive remediation was used with schizo-affective and affective disorders, Anaya et al. (2012) note that effect sizes were comparable with those obtained from the research on schizophrenia. Although limited, these findings suggest that targeting neurocognitive deficits through cognitive remediation can play an effective part in the treatment of BD. This appears particularly promising for the occupational outcomes of BD patients.

In summary, the current review identified a relationship between impaired neurocognitive and occupational functioning in BD. Specifically that neurocognitive impairment in the domains of executive functioning, verbal memory, and processing speed and attention impedes occupational functioning in BD. A comparison of longitudinal and cross-sectional studies indicated that the relationship between neurocognitive impairment and reduced occupational functioning persists over time. Reduced performance on verbal and working memory emerged as important factors for predicting occupational functioning over time, whilst performance on measures of executive functioning were more variable with regards to cross-sectional studies. Clinical variables including residual depression, classification of euthymia and BD diagnoses, medication, premorbid IQ, history of psychosis, illness factors including number of hospitalizations, and co morbid substance abuse and/or dependence were identified as important variables for consideration when evaluating the relationship between neurocognitive and occupational functioning.

There were a number of methodological limitations associated with the variety of neuropsychological assessments employed across studies and the definition and measurement of occupational status that make it difficult to generalise across studies. In lights of these limitations, assessment of neuropsychological function among BD populations is argued to provide important information with regards to occupational functioning, and information for clinicians with regards to rehabilitation options for improving functional outcomes including occupational functioning.

## Appendix

**Table A1:** Details of Neurocognitive and Occupational Research for BD

Study	Sample	Study type	Neurocognitive Domains Measured	Occupational Functioning Measure	Effects of Medication Controlled	Results [Effect Size (ES) *d* or *f^2^*]
Altshuler et al. (2007)	Total (BD I/II) *N* = 213 *M*_Age_ = 43.33 Male = 91%	Cross-sectional study	Structured interview (EXIT); executive functioning (perseveration, response set-switching, generation de novo of stories, generation of word lists, interference)	Employment status; employed vs unemployed	Yes	EXIT composite scores predicted work status (*d* = .77)
Bearden et al. (2011)	Total (EBD I) *N* = 79 *M*_Age_ = 36.6 Male = 53%	Longitudinal study	Neurocognitive assessment battery; processing speed, working memory/attention, episodic memory, visual scanning, executive functioning	Occupational functioning (Life Functioning Questionnaire) Occupationally Recovered vs Unrecovered	Yes	Occupationally Recovered BD participants performed better on: Episodic Memory (CVLT total, immediate, delayed, recognition *d* = .80); Visual Scanning (Span of Apprehension: *d* = .05); Attention/Working Memory (LNS forward and reorder, DSCPT vigilance: *d* = 1.05); Executive Function (WCST category completion and perseverative errors: *d* = .49); Speed of Processing (TMT-A: .19)
Bonnín et al. (2010)	Total *N* = 32 *M*_Age_ = 43.5 Male = 53.1% EBD I (*n* = 24) EBD II (*n* = 8)	Longitudinal study	Neurocognitive assessment battery; estimated IQ, attention, verbal learning and memory, executive functioning	Occupational functioning (Functioning Assessment Short Test)	No	Executive Function (WAIS-III: Digits Backwards) correlated with occupational functioning at 4-year follow-up (*d* = .78).
Bonnín et al. (2014)	Total (EBD I) *N* = 85 *M*_Age_ = 40.11 Male = 48%	Cross-sectional study	Neurocognitive assessment battery; IQ, processing speed index, working memory and attention, verbal learning and memory, executive functioning	Work adjustment; good versus poor	No	Executive functioning (WAIS-III Digits Backwards) predicted poor work adjustment (*f*^2^ = .38)
Bowie et al. (2010)	Total *N* = 291 *M*_Age_ = 49.15 Male = 57% BD I (*n* = 130) SZ (*n* = 161)	Cross-sectional study	Neurocognitive assessment battery; processing speed, verbal declarative and working memory, verbal fluency, attention, executive functioning	Work skills (The Specific Level of Functioning Scale)	No	Adaptive and Social Competence mediated the relationship between Neurocognitive Composite Score and work skills (*f^2^* = .42)
Burdick et al. (2010)	Total (BD I) *N* = 33 *M*_Age_ = 40.2 Male = 54%	Longitudinal study	Neurocognitive assessment battery; processing speed, verbal learning and memory, verbal fluency, accessing of general knowledge, executive functioning	Work disability (Strauss-Carpenter work functioning rating scale)	Yes	Verbal memory (CVLT total) predicted occupational outcome at 15 year follow-up (*d* = .74)
Depp et al. (2012b)	Total (BD I) *N* = 229 *M*_Age_ = 46.85 Male = 50%	Cross-sectional study	Neurocognitive assessment battery; attention, psychomotor speed, verbal and working memory, executive functioning	Occupational status; unemployed versus employed	No	Neurocognitive Composite Score predicted with employment status (*d* = .74)
Dickerson et al. (2004)	Total *N* = 117 *M*_Age_ = 41.4 Male = 30% BD I (*n* = 87) BD II (*n* = 29) BDnos (*n* = 1)	Cross-sectional study	Neurocognitive assessment battery (RBANS); immediate memory, visuospatial and constructional, language, attention, delayed memory	Employment status; no work versus part-time work versus full-time work	Yes	Verbal memory(RBANS list learning and story memory) contributed to employment status (*d* = .82)
Dickerson et al. (2010)	Total *N* = 52 *MAge* = 30.5 Male = 15% BD I (*n* = 38) BD II (*n* = 12) BDnos (*n* = 2)	Prospective longitudinal study	Neurocognitive assessment battery; processing speed, verbal memory, verbal fluency, visual memory, executive functioning, visual spatial ability, premorbid intelligence	Occupational status; full-time employment versus unemployed	No	No relationship emerged between occupational status and neurocognitive functioning.
Gilbert et al. (2010)	Total (BD I) *N* = 148 *M*_Age_ = 44.17 Male = 37%	Post-hoc analysis	Clinical Interview & self report (Bipolar Disorder Visit Form & Mood Spectrum Self-Report Questionnaire); memory and concentration	Employment status; working versus not working	Yes	Self-Reported concentration and memory problems predicted employment status (ES could not be calculated)
Godard et al. (2011)	Total *N* = 30 *M*_Age_ = 47.45 Male = 33% BD I (*n* = 8) BD II (*n* = 6) MDD (*n* = 16)	Cross-sectional study	Neurocognitive assessment battery; visual functions, verbal learning and memory, attention, executive functioning	Work functioning (Longitudinal Interval Follow-up Evaluation-Range of Impaired Functioning Tool)	No	Attention (CogitEx II Simple RT; *d* = 1.14), Executive Function (D-KEFS Verbal Fluency Test *d =* 1.11) and verbal memory (CVLT-II retrieval *d* = 1.12) associated with work functioning
Martínez-Arán et al. (2004)	Total *N* = 138 *M*_Age_ = 41.1 Male = 42% BDD (*n* = 30) BDMH (*n* = 34) EBD (*n* = 44) HC (*n* = 30)	Cross-sectional study	Neurocognitive assessment battery; estimated premorbid IQ, verbal learning and memory, nonverbal learning and memory, attention/ concentration and mental tracking, executive functioning	Occupational functioning; good versus poor	No	Verbal memory (CVLT immediate and delayed) and executive functioning (COWAT) associated with occupational functioning (ES could not be calculated)
Martínez-Arán et al. (2007)	Total *N* = 112 *M*_Age_ = 39 Male = 38% EBD I (*n* = 59) EBD II (*n* = 18) HC (*n* = 35)	Cross-sectional study	Neurocognitive assessment battery; estimated premorbid IQ, verbal learning and memory, executive functioning	Occupational adaptation; good versus low	Yes	Verbal memory (CVLT immediate and free recall); Executive Function (COWAT; TMT-B) associated with occupational adaptation. (ES could not be calculated)
Mora et al. (2013)	Total *N* = 54 *M*_Age_ = 41.55 Male = 48% EBD (*n* = 28) HC (*n* = 26)	Longitudinal study	Neurocognitive assessment battery; processing speed, attention, verbal memory, visual memory, executive functioning	Occupational status; active versus inactive versus retired	No	Attention (CPT-II hit RT), Verbal Memory (CVLT immediate and delayed recall), Executive Function (TMT-B; Stroop Colour Word Test) associated with occupational functioning at 6 year follow-up (ES could not be calculated)
Mur et al. (2008)	Total *N* = 66 *M*_Age_ = 41.2 Male = 51.5% EBD (*n* = 33) HC (*n* = 33)	Longitudinal study	Neurocognitive assessment battery; processing speed, attention, verbal memory, visual memory, executive functioning	Occupational status; active versus inactive versus retired	No	Processing speed (TMT-A: *d* = 1.15) and Executive Function (Stroop colour and Word Test: *d* = .87) associated with work status at 2 year follow-up
Mur et al. (2009)	Total *N* = 44 *M*_Age_ = 42.9 Male = 50% EBD I (*n* = 30) EBD II (*n*= 14)	Cross-sectional study	Neurocognitive assessment battery; processing speed, verbal memory, visual memory, attention, inhibition, executive functioning	Occupational status; active versus inactive	Yes	Significantly greater Executive Function (Stroop Colour and Word Test:*d* =.80), processing speed (TMT-A: *d* = .77) for active vs inactive participants
Murtagh et al. (2010)	Total *N* = 77 *M*_Age_ = 42 Male = 63% BD (*n* = 13) SZ (*n* = 45) SA (*n* = 17) PD (*n* = 1) DD (*n* = 1)	Post hoc analysis	Neurocognitive assessment battery; current IQ, episodic memory, face recognition, working memory, attention, social awareness, word fluency	Work status; worked in the last 3 years	No	Executive functioning (WMS LNS) associated with work status (ES could not be calculated)
O'Shea et al. (2010)	Total *N* = 58 *M*_Age_ = 53 Male = 48.2% EBD (*n* = 29) HC (*n* = 29)	Cross-sectional study	Ecologically valid cognitive test battery; attention, memory, executive functioning.	Occupational functioning (The Social and Occupational Functional Assessment Scale)	No	Attention (TEA) was associated with occupational functioning (ES could not be calculated)
Ryan et al. (2013)	Total *N* = 299 Mean age = 38.43 Male = 57% EBD (*n* = 156) HC (*n* = 143)	Cross-sectional study	Neurocognitive assessment battery; visual memory, auditory memory, emotion processing, fine motor dexterity, verbal fluency & processing speed, conceptual reasoning and set-shifting, processing speed with interference resolution, inhibitory control	Work status; working versus not working	Yes	Verbal Fluency (COWAT: *d* = .38) and Processing Speed Intereference Resolution (Stroop Colour and Word test: *d* = .11) associated with work status
Schoeyen et al. (2013)	Total *N* = 226 *M*_Age_ = 33.9 Male = 38% BD I (*n* = 144) BD II (*n* = 70) BDnos (*n* = 12)	Cross-sectional study	Neurocognitive assessment battery; IQ, premorbid intellectual functioning	Receipt of disability benefit	No	Occupational outcome not associated with premorbid, current, or decline in, IQ
Tabarés-Seisdedos et al. (2008)	Total *N* = 115 *M*Age = 38.7 Male = 62% BD I (*n* = 43) SZ (*n* = 47) HC (*n* = 25)	Longitudinal study	Neurocognitive assessment battery; executive functioning and problem solving, verbal working memory, verbal memory, visual memory, visual-motor processing/speed of processing, vigilance, motor speed, language/vocabulary	Occupational adaptation; good versus low	No	Neurocognitive Composite Score strongest predictor of occupational adaptation at one year follow-up (*d* = .57)
Torrent et al. (2006)	Total *N* = 106 *M*_Age_ = 40.9 Male = 41% EBD I (*n* = 38) EBD II (*n* = 33) HC (*n* = 35)	Cross-sectional study	Neurocognitive assessment battery; estimated premorbid IQ, verbal learning and memory, attention/ concentration and mental tracking, executive functioning	Occupational adaptation; good versus low	No	Executive functioning (TMT-B) predicted occupational adaptation in BD II (ES could not be calculated)
Wingo et al. (2010)	Total *N* = 65 *M*_Age_ = 40.1 Male = 50.8% EBD I (*n* = 42) EBD II (*n* = 23)	Cross-sectional study	Neurocognitive assessment battery; estimated premorbid IQ, verbal learning and memory, attention and concentration, executive functioning	Occupational functioning (Vocational Status Index)	Yes	No relationship between neurocognitive functioning and occupational functioning

*Note.* Bipolar Disorder (BD), Bipolar Disorder Depressed (BDD), Bipolar Disorder Manic or Hypomanic (BDMH), Bipolar Disorder not otherwise specified (BDnos), Bipolar Disorder with Psychosis (BD I wP), Bipolar Disorder without Psychosis (BD I woP), Euthymic Bipolar Disorder (EBD), Major Depressive Disorder (MDD), Schizophrenia (SZ), Schizoaffective Disorder (SA), Psychotic Depression (PD), Delusional Disorder (DD), Healthy Controls (HC), California Verbal Learning Test (CVLT), Cognitive and Executive Function (Cogit Ex II), Controlled Oral Word Association Test (COWAT), Continuous Performance Test (CPT-II), Degraded Stimulus Continuous Performance Test (DSCPT), Delis-Kaplan Executive Function System (D-KEFS), Executive Interview (EXIT), Repeatable Battery for the Assessment of Neuropsychological Status (RBANS), Test of Everyday Attention (TEA),Trail Making Test Part A/B (TMT-A, TMT-B), Wechsler Adult Intelligence Scale (WAIS-III), Wechsler Memory Scale (WMS), Wisconsin Card Sorting Test (WCST).

## References

[r1] Altshuler L. Tekell J. Biswas K. Kilbourne A. M. Evans D. Tang D. Bauer M. S. (2007). Executive function and employment status among veterans with bipolar disorder. Psychiatric Services, 58(11), 1441–1447. doi:. 10.1176/ps.2007.58.11.1441 17978254

[r2] Anaya C. Martínez-Arán A. Ayuso-Mateos J. L. Wykes T. Vieta E. Scott J. (2012). A systematic review of cognitive remediation for schizo-affective and affective disorders. Journal of Affective Disorders, 142, 13–21. 10.1016/j.jad.2012.04.020 22840620

[r3] American Psychiatric Association. (2013). *Diagnostic and statistical manual of mental disorders* (5th ed.). Arlington, VA, USA: American Psychiatric Association.

[r4] Anderson I. M. Haddad P. M. Scott J. (2012). Bipolar disorder. BMJ: British Medical Journal, 345, e8508. 10.1136/bmj.e8508 23271744

[r5] Andreou C. Bozikas V. P. (2013). The predictive significance of neurocognitive factors for functional outcome in bipolar disorder. Current Opinion in Psychiatry, 26(1), 54–59. 10.1097/YCO.0b013e32835a2acf 23154642

[r6] Balanzá-Martínez V. Selva G. Martínez-Arán A. Prickaerts J. Salazar J. González-Pinto A. Tabarés-Seisdedos R. (2010). Neurocognition in bipolar disorders – A closer look at comorbidities and medications. European Journal of Pharmacology, 626, 87–96. 10.1016/j.ejphar.2009.10.018 19836378

[r7] Bauer M. S. Kirk G. F. Gavin C. Williford W. O. (2001). Determinants of functional outcome and healthcare costs in bipolar disorder: A high-intensity follow-up study. Journal of Affective Disorders, 65(3), 231–241. 10.1016/S0165-0327(00)00247-0 11511403

[r8] Baune B. T. Li X. Beblo T. (2013). Short- and long-term relationships between neurocognitive performance and general function in bipolar disorder. Journal of Clinical and Experimental Neuropsychology, 35(7), 759–774. doi:. 10.1080/13803395.2013.824071 23944232

[r9] Bearden C. E. Shih V. H. Green M. F. Gitlin M. Sokolski K. N. Levander E. Altshuler L. L. (2011). The impact of neurocognitive impairment on occupational recovery of clinically stable patients with bipolar disorder: A prospective study. Bipolar Disorders, 13(4), 323–333. doi:. 10.1111/j.1399-5618.2011.00928.x 21843272 PMC3157039

[r10] Bonnín C. M. Martínez-Arán A. Torrent C. Pacchiarotti I. Rosa A. R. Franco C. Vieta E. (2010). Clinical and neurocognitive predictors of functional outcome in bipolar euthymic patients: A long-term, follow-up study. Journal of Affective Disorders, 121(1-2), 156–160. 10.1016/j.jad.2009.05.014 19505727

[r11] Bonnín C. M. Torrent C. Goikolea J. M. Reinares M. Solé B. Valentí M. Vieta E. (2014). The impact of repeated manic episodes and executive dysfunction on work adjustment in bipolar disorder. European Archives of Psychiatry and Clinical Neuroscience, 264(3), 247–254. doi:. 10.1007/s00406-013-0431-2 23912643

[r12] Bora E. Yücel M. Pantelis C. Berk M. (2011). Meta-analytic review of neurocognition in bipolar II disorder. Acta Psychiatrica Scandinavica, 123(3), 165–174. doi:. 10.1111/j.1600-0447.2010.01638.x 21092023

[r13] Bowie C. R. Pulver A. E. Depp C. McGrath J. A. Wolyniec P. Mausbach B. T. Harvey P. D. (2010). Prediction of real-world functional disability in chronic mental disorders: A comparison of schizophrenia and bipolar disorder. The American Journal of Psychiatry, 167(9), 1116–1124. doi:. 10.1176/appi.ajp.2010.09101406 20478878 PMC3694770

[r14] Burdick K. E. Endick C. J. Goldberg J. F. (2005). Assessing cognitive deficits in bipolar disorder: Are self-reports valid? Psychiatry Research, 136(1), 43–50. 10.1016/j.psychres.2004.12.009 16024090

[r15] Burdick K. E. Goldberg J. F. Harrow M. (2010). Neurocognitive dysfunction and psychosocial outcome in patients with bipolar I disorder at 15-year follow-up. Acta Psychiatrica Scandinavica, 122(6), 499–506. doi:. 10.1111/j.1600-0447.2010.01590.x 20637012 PMC2980854

[r16] Cahill C. M. Green M. J. Jairam R. Malhi G. S. (2007). Do cognitive deficits in juvenile bipolar disorder persist into adulthood? The Journal of Nervous and Mental Disease, 195(11), 891–896. doi:. 10.1097/NMD.0b013e318159288b 18000450

[r17] Dean B. B. Gerner D. Gerner R. H. (2004). A systematic review evaluating health-related quality of life, work impairment, and health-care costs and utilization in bipolar disorder. Current Medical Research and Opinion, 20(2), 139–154. 10.1185/030079903125002801 15006007

[r18] Deckersbach T. Nierenberg A. A. Kessler R. Lund H. G. Ametrano R. M. Sachs G. Dougherty D. (2010). Cognitive rehabilitation for bipolar disorder: An open trial for employed patients with residual depressive symptoms. CNS Neuroscience & Therapeutics, 16, 298–307. 10.1111/j.1755-5949.2009.00110.x 19895584 PMC2888654

[r19] Delaloye C. de Bilbao F. Moy G. Baudois S. Weber K. Campos L. Gold G. (2009). Neuroanatomical and neuropsychological features of euthymic patients with bipolar disorder. The American Journal of Geriatric Psychiatry, 17(12), 1012–1021. 10.1097/JGP.0b013e3181b7f0e2 20104058

[r20] Demant K. M. Almer G. M. Vinberg M. Vedel Kessing L. Miskowiak K. W. (2013). Effects of cognitive remediation on cognitive dysfunction in partially or fully remitted patients with bipolar disorder: Study protocol for a randomized controlled trial. Trials, 14, 378. 10.1186/1745-6215-14-378 24206639 PMC4226194

[r21] Depp C. A. Mausbach B. T. Bowie C. Wolyniec P. Thornquist M. H. Luke J. R. Patterson T. L. (2012 b). Determinants of occupational and residential functioning in bipolar disorder. Journal of Affective Disorders, 136, 812–818. 10.1016/j.jad.2011.09.035 22129770 PMC3639009

[r22] Depp C. A. Mausbach B. T. Harmell A. L. Savla G. N. Bowie C. R. Harvey P. D. Patterson T. L. (2012 a). Meta-analysis of the association between cognitive abilities and everyday functioning in bipolar disorder. Bipolar Disorders, 14(3), 217–226. doi:. 10.1111/j.1399-5618.2012.01011.x 22548895 PMC3396289

[r23] Dickerson F. B. Boronow J. J. Stallings C. R. Origoni A. E. Cole S. Yolken R. H. (2004). Association between cognitive functioning and employment status of persons with bipolar disorder. Psychiatric Services, 55, 54–58. doi:. 10.1176/appi.ps.55.1.54 14699201

[r24] Dickerson F. B. Origoni A. E. Stallings C. R. Khushalani S. Dickinson D. Medoff D. (2010). Occupational status and social adjustment six months after hospitalisation early in the course of bipolar disorder: A prospective study. Bipolar Disorders, 12, 10–20. 10.1111/j.1399-5618.2009.00784.x 20148863

[r25] Eklund M. Hansson L. Ahlqvist C. (2004). The importance of work as compared to other forms of daily occupations for wellbeing and functioning among persons with long-term mental illness. Community Mental Health Journal, 40(5), 465–477. 10.1023/B:COMH.0000040659.19844.c2 15529479

[r26] Elgie R. Morselli P. L. (2007). Social functioning in bipolar patients: The perception and perspective of patients, relatives and advocacy organizations – A Review. Bipolar Disorders, 9, 144–157. 10.1111/j.1399-5618.2007.00339.x 17391357

[r27] Fisher L. J. Goldney R. D. Dal Grande E. Taylor A. W. Hawthorne G. (2007). Bipolar disorders in Australia: A population-based study of excess costs. Social Psychiatry and Psychiatric Epidemiology, 42, 105–109. 10.1007/s00127-006-0133-4 17080320

[r28] Gilbert A. M. Olino T. M. Houck P. Fagiolini A. Kupfer D. J. Frank E. (2010). Self-reported cognitive problems predict employment trajectory in patients with bipolar I disorder. Journal of Affective Disorders, 124(3), 324–328. doi:. 10.1016/j.jad.2009.11.012 19942294 PMC2888870

[r29] Gilbert E. Marwaha S. (2013). Predictors of employment in bipolar disorder: A systematic review. Journal of Affective Disorders, 145(2), 156–164. 10.1016/j.jad.2012.07.009 22877965

[r30] Godard J. Grondin S. Baruch P. Lafleur M. F. (2011). Psychosocial and neurocognitive profiles in depressed patients with major depressive disorder and bipolar disorder. Psychiatry Research, 190(2-3), 244–252. 10.1016/j.psychres.2011.06.014 21764461

[r31] Harvey P. D. Wingo A. P. Burdick K. E. Baldessarini R. J. (2010). Cognition and disability in bipolar disorder: Lessons from schizophrenia research. Bipolar Disorders, 12, 364–375. 10.1111/j.1399-5618.2010.00831.x 20636633

[r32] Keck P. E. (2004). Defining and improving response to treatment in patients with bipolar disorder. The Journal of Clinical Psychiatry, 65(Suppl. 15), 25–29. 15554793

[r33] Kurtz M. M. Gerraty R. T. (2009). A meta-analytic investigation of neurocognitive deficits in bipolar illness: Profile and effects of clinical state. Neuropsychology, 23(5), 551–562. doi:. 10.1037/a0016277 19702409 PMC2804472

[r34] Levy B. Manove E. (2012). Functional outcome in bipolar disorder: The big picture. Depression Research and Treatment, 2012, 949248. 10.1155/2012/949248 21961062 PMC3180778

[r35] Levy B. Medina A. M. Weiss R. D. (2013). Cognitive and psychosocial functioning in bipolar disorder with and without psychosis during early remission from an acute mood episode: A comparative longitudinal study. Comprehensive Psychiatry, 54(6), 618–626. doi:. 10.1016/j.comppsych.2012.12.018 23357126 PMC4076957

[r36] MacQueen G. M. Young L. T. Joffe R. T. (2001). A review of psychosocial outcome in patients with bipolar disorder. Acta Psychiatrica Scandinavica, 103(3), 163–170. doi:. 10.1034/j.1600-0447.2001.00059.x 11240572

[r37] Martínez-Arán A. Vieta E. Torrent C. Sanchez-Moreno J. Goikolea J. M. Salamero M. Ayuso-Mateos C. (2007). Functional outcome in bipolar disorder: The role of clinical and cognitive factors. Bipolar Disorders, 9(1-2), 103–113. doi:. 10.1111/j.1399-5618.2007.00327.x 17391354

[r38] Martínez-Arán A. Salamero M. Vieta E. Reinares M. Colom F. Torrent C. Comes M. (2004). Cognitive function across manic or hypomanic, depressed, and euthymic states in bipolar disorder. The American Journal of Psychiatry, 161(2), 262–270. doi:. 10.1176/appi.ajp.161.2.262 14754775

[r39] Martino D. J. Marengo E. Igoa A. Scápola M. Ais E. D. Perinot L. Strejilevich S. A. (2009). Neurocognitive and symptomatic predictors of functional outcome in bipolar disorders: A prospective 1 year follow-up study. Journal of Affective Disorders, 116(1-2), 37–42. doi:. 10.1016/j.jad.2008.10.023 19033081

[r40] Marwaha S. Durrani A. Singh S. (2013). Employment outcomes in people with bipolar disorder: A systematic review. Acta Psychiatrica Scandinavica, 128(3), 179–193. doi:. 10.1111/acps.12087 23379960

[r41] Michalak, E. E., & Murray, G. (2010). A clinician's guide to psychosocial functioning and quality of life in bipolar disorder. In A. Young, N. Ferrier, & E. E. Michalak (Eds.), *Practical management of bipolar disorder* (pp. 163-174). Cambridge, United Kingdom: Cambridge University Press.

[r42] Michalak E. E. Yatham L. N. Lam R. W. (2005). Quality of life in bipolar disorder: A review of the literature. Health and Quality of Life Outcomes, 3, 72. 10.1186/1477-7525-3-72 16288650 PMC1325049

[r43] Michalak E. E. Yatham L. N. Maxwell V. Hale S. Lam R. W. (2007). The impact of bipolar disorder upon work functioning: A qualitative analysis. Bipolar Disorders, 9(1-2), 126–143. doi:. 10.1111/j.1399-5618.2007.00436.x 17391356

[r44] Mora E. Portella M. J. Forcada I. Vieta E. Mur M. (2013). Persistence of cognitive impairment and its negative impact on psychosocial functioning in lithium treated, euthymic bipolar patients: A 6-year follow-up study. Psychological Medicine, 43, 1187–1196. doi:. 10.1017/S0033291712001948 22935452

[r45] Mur M. Portella M. J. Martínez-Arán A. Pifarre J. Vieta E. (2008). Long-term stability of cognitive impairment in bipolar disorder: A 2-year follow-up study of lithium treated euthymic bipolar patients. The Journal of Clinical Psychiatry, 69, 712–719. 10.4088/JCP.v69n0504 18435565

[r46] Mur M. Portella M. J. Martínez-Arán A. Pifarre J. Vieta E. (2009). Influence of clinical and neuropsychological variables on the psychosocial and occupational outcome of remitted bipolar patients. Psychopathology, 42, 148–156. doi:. 10.1159/000207456 19276630

[r47] Murtagh A. Hurley A. L. Kinsella A. Corvin A. Donohoe G. Gill M. Murphy K. C. (2010). The letter-number sequencing test and its association with potential to work among people with psychotic illness. European Psychiatry, 25, 101–104. doi:. 10.1016/j.eurpsy.2009.06.004 19720503

[r48] Nordt C. Müller B. Rössler W. Lauber C. (2007). Predictors and course of vocational status, income, and quality of life in people with severe mental illness: A naturalistic study. Social Science & Medicine, 65(7), 1420–1429. doi:. 10.1016/j.socscimed.2007.05.024 17583402

[r49] O'Shea R. Poz R. Michael A. Berrios G. E. Evans J. J. Rubinsztein J. S. (2010). Ecologically valid cognitive tests and everyday functioning in euthymic bipolar disorder patients. Journal of Affective Disorders, 125(1-3), 336–340. doi:. 10.1016/j.jad.2009.12.012 20609481

[r50] Robinson L. J. Thompson J. M. Gallagher P. Goswami U. Young A. H. Ferrier I. N. Moore P. B. (2006). A meta-analysis of cognitive deficits in euthymic patients with bipolar disorder. Journal of Affective Disorders, 93(1-3), 105–115. doi:. 10.1016/j.jad.2006.02.016 16677713

[r51] Rosa A. R. Reinares M. Franco C. Comes M. Torrent C. Sánchez-Moreno J. Vieta E. (2009). Clinical predictors of functional outcome of bipolar patients in remission. Bipolar Disorders, 11(4), 401–409. 10.1111/j.1399-5618.2009.00698.x 19500093

[r52] Ryan K. A. Vederman A. C. Kamali M. Marshall D. Weldon A. L. McInnis M. G. Langenecker S. A. (2013). Emotion perception and executive functioning predict work status in euthymic bipolar disorder. Psychiatry Research, 210, 472–478. doi:. 10.1016/j.psychres.2013.06.031 23870493

[r53] Sanchez-Moreno J. Martínez-Arán A. Tabarés-Seisdedos R. Torrent C. Vieta E. Ayuso-Mateos J. L. (2009). Functioning and disability in bipolar disorder: An extensive review. Psychotherapy and Psychosomatics, 78(5), 285–297. doi:. 10.1159/000228249 19602917

[r54] Schoeyen H. K. Melle I. Sundet K. Aminoff S. R. Hellvin T. Auestad B. H. Andreassen O. A. (2013). Occupational outcome in bipolar disorder is not predicted by premorbid functioning and intelligence. Bipolar Disorders, 15(3), 294–305. doi:. 10.1111/bdi.12056 23527993

[r55] Shippee N. D. Shah N. D. Williams M. D. Moriarty J. P. Frye M. A. Ziegenfuss J. Y. (2011). Differences in demographic composition and in work, social, and functional limitations among the populations with unipolar depression and bipolar disorder: Results from a nationally representative sample. Health and Quality of Life Outcomes, 9, 90. 10.1186/1477-7525-9-90 21995725 PMC3207868

[r56] Simonsen C. Sundet K. Vaskinn A. Ueland T. Romm K. L. Hellvin T. Andreassen O. A. (2010). Psychosocial function in schizophrenia and bipolar disorder: Relationship to neurocognition and clinical symptoms. Journal of the International Neuropsychological Society, 16(5), 771–783. doi:. 10.1017/S1355617710000573 20509984

[r57] Sole B. Bonnin C. M. Torrent C. Martínez-Arán A. Popovic D. Tabares-Seisdedos R. Vieta E. (2012). Neurocognitive impairment across the bipolar spectrum. CNS Neuroscience & Therapeutics, 18(3), 194–200. doi:. 10.1111/j.1755-5949.2011.00262.x 22128808 PMC6493522

[r58] Tabarés-Seisdedos R. Balanzá-Martínez V. Sánchez-Moreno J. Martínez-Arán A. Salazar-Fraile J. Selva-Vera G. Vieta E. (2008). Neurocognitive and clinical predictors of functional outcome in patients with schizophrenia and bipolar I disorder at one-year follow-up. Journal of Affective Disorders, 109(3), 286–299. 10.1016/j.jad.2007.12.234 18289698

[r59] Tohen M. Hennen J. Zarate C. M. Baldessarini R. J. Strakowski S. M. Stoll A. L. Cohen B. M. (2000). Two-year syndromal and functional recovery in 219 cases of first-episode major affective disorder with psychotic features. The American Journal of Psychiatry, 157(2), 220–228. 10.1176/appi.ajp.157.2.220 10671390

[r60] Torrent C. del Mar Bonnin C. Martínez-Arán A. Valle J. Amann B. L. González-Pinto A. Crespo J. M. Vieta E. (2013). Efficacy of functional remediation in bipolar disorder: A multicenter randomized controlled study. The American Journal of Psychiatry, 170, 852–859. 10.1176/appi.ajp.2012.12070971 23511717

[r61] Torrent C. Martínez-Arán A. Daban C. Sánchez-Moreno J. Comes M. Goikolea J. M. Vieta E. (2006). Cognitive impairment in bipolar II disorder. The British Journal of Psychiatry, 189(3), 254–259. doi:. 10.1192/bjp.bp.105.017269 16946361

[r62] Torres I. J. Boudreau V. G. Yatham L. N. (2007). Neuropsychological functioning in euthymic bipolar disorder: A meta-analysis. Acta Psychiatrica Scandinavica, 116, 17–26. doi:. 10.1111/j.1600-0447.2007.01055.x 17688459

[r63] Torres I. J. Solé B. Vieta E. Martínez-Arán A. (2012). Neurocognitive impairment in the bipolar spectrum. Neuropsychiatry, 2(1), 43–55. doi:. 10.2217/npy.12.3 PMC6493522 22128808

[r64] Tse S. Chan S. Ng K. L. Yatham L. N. (2014). Meta-analysis of predictors of favorable employment outcomes among individuals with bipolar disorder. Bipolar Disorders, 16(3), 217–229. doi:. 10.1111/bdi.12148 24219657

[r65] Waghorn G. Lloyd C. (2005). The employment of people with mental illness. Advances in Mental Health, 4(2), 129–171. 10.5172/jamh.4.2.129

[r66] Wingo A. P. Baldessarini R. J. Holtzheimer P. E. Harvey P. D. (2010). Factors associated with functional recovery in bipolar disorder patients. Bipolar Disorders, 12(3), 319–326. doi:. 10.1111/j.1399-5618.2010.00808.x 20565439 PMC3749090

[r67] Wingo A. P. Harvey P. D. Baldessarini R. J. (2009). Neurocognitive impairment in bipolar disorder patients: Functional implications. Bipolar Disorders, 11(2), 113–125. 10.1111/j.1399-5618.2009.00665.x 19267694

[r68] Yatham L. N. Torres I. J. Malhi G. S. Frangou S. Glahn D. C. Bearden C. E. Goldberg J. F. (2010). The International Society for Bipolar Disorders–Battery for Assessment of Neurocognition (ISBD-BANC). Bipolar Disorders, 12(4), 351–363. 10.1111/j.1399-5618.2010.00830.x 20636632

[r69] Zimmerman M. Galione J. N. Chelminski I. Young D. Dalrymple K. Ruggero C. J. (2010). Sustained unemployment in psychiatric outpatients with bipolar disorder: Frequency and association with demographic variables and comorbid disorders. Bipolar Disorders, 12(7), 720–726. doi:. 10.1111/j.1399-5618.2010.00869.x 21040289

[r70] Zutshi A. Eckert K. A. Hawthorne G. Taylor A. W. Goldney R. D. (2011). Changes in the prevalence of bipolar disorders between 1998 and 2008 in an Australian population. Bipolar Disorders, 13, 182–188. doi:. 10.1111/j.1399-5618.2011.00907.x 21443572

